# Google Trends Insights Into Reduced Acute Coronary Syndrome Admissions During the COVID-19 Pandemic: Infodemiology Study

**DOI:** 10.2196/20426

**Published:** 2020-08-24

**Authors:** Conor Senecal, Rajiv Gulati, Amir Lerman

**Affiliations:** 1 Mayo Clinic Rochester, MN United States

**Keywords:** Google Trends, acute coronary syndrome, coronary heart disease, online search, internet, trend, COVID-19, heart, cardiovascular

## Abstract

**Background:**

During the coronavirus disease (COVID-19) pandemic, a reduction in the presentation of acute coronary syndrome (ACS) has been noted in several countries. However, whether these trends reflect a reduction in ACS incidence or a decrease in emergency room visits is unknown. Using Google Trends, queries for chest pain that have previously been shown to closely correlate with coronary heart disease were compared with searches for myocardial infarction and COVID-19 symptoms.

**Objective:**

The current study evaluates if search terms (or topics) pertaining to chest pain symptoms correlate with the reported decrease in presentations of ACS.

**Methods:**

Google Trends data for search terms “chest pain,” “myocardial infarction,” “cough,” and “fever” were obtained from June 1, 2019, to May 31, 2020. Related queries were evaluated for a relationship to coronary heart disease.

**Results:**

Following the onset of the COVID-19 pandemic, chest pain searches increased in all countries studied by at least 34% (USA *P*=.003, Spain *P*=.007, UK *P*=.001, Italy *P*=.002), while searches for myocardial infarction dropped or remained unchanged. Rising searches for chest pain included “coronavirus chest pain,” “home remedies for chest pain,” and “natural remedies for chest pain.” Searches on COVID-19 symptoms (eg, cough, fever) rose initially but returned to baseline while chest pain–related searches remained elevated throughout May.

**Conclusions:**

Search engine queries for chest pain have risen during the pandemic as have related searches with alternative attribution for chest pain or home care for chest pain, suggesting that recent drops in ACS presentations may be due to patients avoiding the emergency room and potential treatment in the midst of the COVID-19 pandemic.

## Introduction

Amid the coronavirus disease (COVID-19) pandemic, a reduction in ST-elevation myocardial infarction (STEMI) activations and presentation to the emergency room across the United States has been noted [1]. In a large diverse community setting in California, acute coronary syndrome (ACS) admissions fell by nearly half [2]. Similarly, in the United Kingdom, there has been a sharp decline in emergency department (ED) visits for myocardial ischemia [3], and in Italy, ACS admissions were noted to fall dramatically [4]. The extent to which these observations are due to a true reduction in ACS incidence or avoidance of hospitals remains unknown. Even in the setting of a pandemic, delays in diagnosis and treatment for ACS is associated with increased mortality [5]. Our previous work has shown a consistent correlation between search engine queries for chest pain and similar symptoms with the prevalence of coronary artery disease [6]. COVID-19–related changes in search patterns pertaining to health care have already been reported [7,8]. Given that chest pain is not a common symptom of COVID-19, this relationship would be expected to be consistent during a pandemic [9]. We investigated changes in chest pain search frequency before and after the onset of the COVID-19 pandemic and compared this to myocardial infarction search frequency, as well as changes in search frequency for symptoms of COVID-19.

## Methods

The use of Google Trends search data in health research has been previously described [10]. Results provided by this service are reported as relative search volume (RSV). Each data point is divided by the total searches pertaining to the geographic area and time range it represents to compare relative popularity. The resulting numbers are then scaled on a range of 0 to 100 based on a topic’s proportion to all searches on all topics. Different regions or time frames that show the same RSV for a term do not always have the same total search volumes. For example, a given RSV for “chest pain” in the United States is only directly comparable to an RSV for Italy if the searches were performed together, and even in this circumstance the frequency represents the popularity of a search per the total volume of searches in the geographic area as opposed to absolute search volume. Due to this limitation, comparisons of RSV were only made within single queries (ie, searched together). Since RSV is reported based on an adjusted 0-100 scale for each query, comparisons of RSV from separate queries are not useful. Therefore, statistical comparisons used in this analysis were carried out between data from single queries (Table 1). Search topics were used in this analysis as they encompass synonyms, similar terms, and results from multiple languages for the topic searched as opposed to search terms, which only returns results on the exact term used. For example, the topic “myocardial infarction” includes the terms “heart attack,” “symptoms of heart attack,” or “infarto de miocardio.” More information about Google Trends search results is available online [11]. The URL for each query made in Google Trends has been included in Table 1 to provide the exact search terminology used.

We queried “chest pain” and “myocardial infarction” separately as topics from June 1, 2019, to May 31, 2020, in the following search regions: the United States, the United Kingdom, Spain, and Italy. As only five queries are simultaneously allowed, we then created a search with the “chest pain” topic and the “myocardial infarction” topic for the United States during the same time frame to allow for a direct comparison. We defined the pre–COVID-19 and post–COVID-19 time frames as before and after March 1, the day following the first confirmed US death from COVID-19. Additionally, we separately searched “cough” and “fever” symptoms associated with COVID-19 for the United States during the same time frame. Pre– and post–COVID-19 mean RSVs were compared using paired *t* tests with an alpha of .05 and 95% CIs.

Google Trends also provides “related queries” to searches made by the same users and separates these into those that have increased the most compared to a previous time period (“rising”) and those most commonly searched (“top”). These search terms were evaluated for any relationship to reduced ACS presentations. Queries were made for “chest pain,” “chest pain – corona,” and “chest pain – COVID” from March 15 to April 15, the month-long period with the highest searches for chest pain in the post–COVID-19 analysis, with the latter two queries made in an attempt to isolate searches unrelated to COVID-19. For these searches, search terms were used as opposed to topics to allow for the removal of COVID-19–associated terminology.

**Table 1:** Google Trends queries used for data acquisition.

Search terms	Duration	Geography	URL
Chest pain (topic)	June 1, 2019 - May 30, 2020	United States, Spain, Italy, United Kingdom	https://bit.ly/3dPisGi
Myocardial infarction (topic)	June 1, 2019 - May 30, 2020	United States, Spain, Italy, United Kingdom	https://bit.ly/3dV5EOO
Fever (topic) + cough (topic)	June 1, 2019 - May 30, 2020	United States	https://rb.gy/wnbvci
Chest pain (topic) + myocardial infarction (topic)	June 1, 2019 - May 30, 2020	United States	https://bit.ly/37mL8E5
Chest pain (topic), chest pain – COVID (term), chest pain – corona (term)	March 15, 2020 - April 15, 2020	United States	https://bit.ly/30LdiHy

## Results

**Figure 1:**
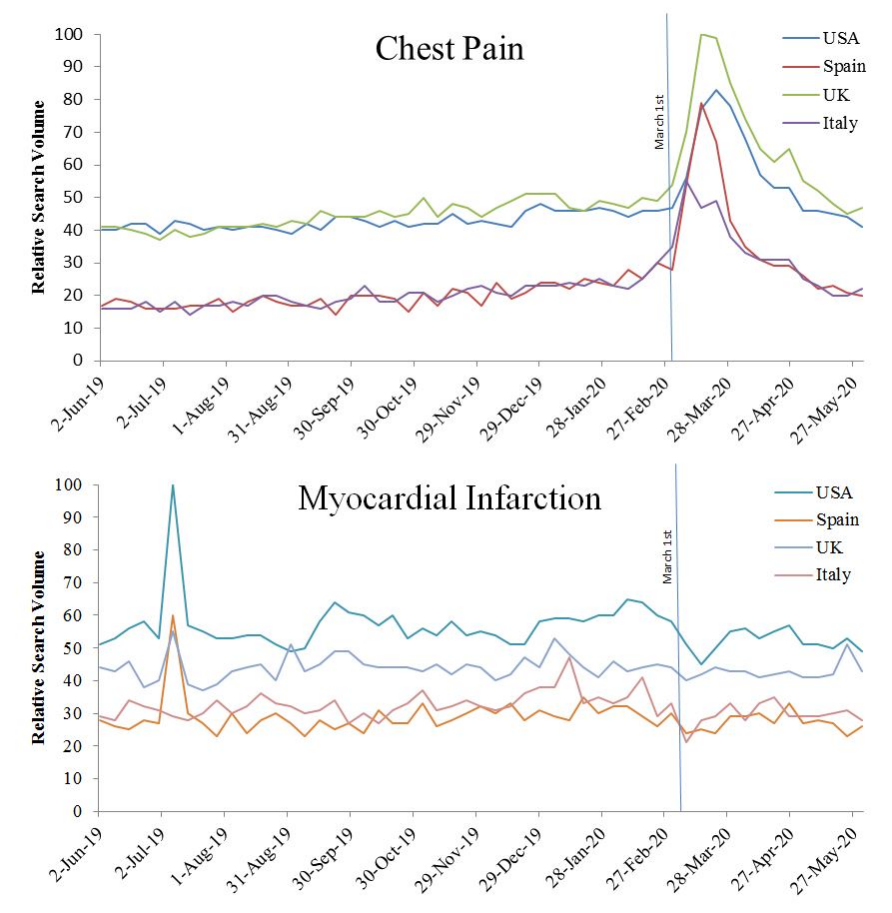
Relative daily search frequency of chest pain (top) and myocardial infarction (bottom) from June 1, 2020, to May 31, 2020, in the United States, United Kingdom, Spain, and Italy.

Search frequency for chest pain and myocardial infarction topics from June 1, 2019, to May 31, 2020, in the United States, Spain, United Kingdom, and Italy are shown in Figure 1. From June 1, 2019 to March 1, 2020, search frequency for chest pain varied between the four countries evaluated but showed little variation. From March 1 to May 31, 2020, all four countries evaluated saw a rise in searches for chest pain compared to the period prior to March 1, 2020 (USA *P*=.003, Spain *P*=.007, UK *P*=.001, Italy *P*=.002). All countries had at least a 34% rise in searches, with Spain seeing the largest increase at 84%. RSV for the topic myocardial infarction also varied in the four countries. The United States and Italy exhibited a significant drop in searches related to myocardial infarction from baseline, of 9% (57% to 52%, *P*=.001) and 10% (33% to 29%, *P*=.008), respectively, between March 1 to May 31, 2020. Similarly, searches for myocardial infarction fell in the United Kingdom and Spain but did not reach statistical significance. In the United States, the topic myocardial infarction was searched 1.4x as often as the topic chest pain before March 1, 2020; after that date it was searched 0.91x as often. “Chest pain – COVID” and “chest pain – corona” both showed an increase in search volume from pre–COVID-19 to post–COVID-19 time frames as well.

Rising searches associated with chest pain searches are shown in Table 2 and included “is chest pain a symptom of COVID” and “is chest pain a sign of coronavirus.” Rising searches unrelated to COVID-19 included “home remedies for chest pain” and “natural remedies for chest pain,” which both had a >41x increase, along with “persistent pain or pressure in the chest,” which had a 6x increase. Top related queries for all three queries were similar, with top two being “pain in chest” and “coronavirus chest pain.”

**Figure 2:**
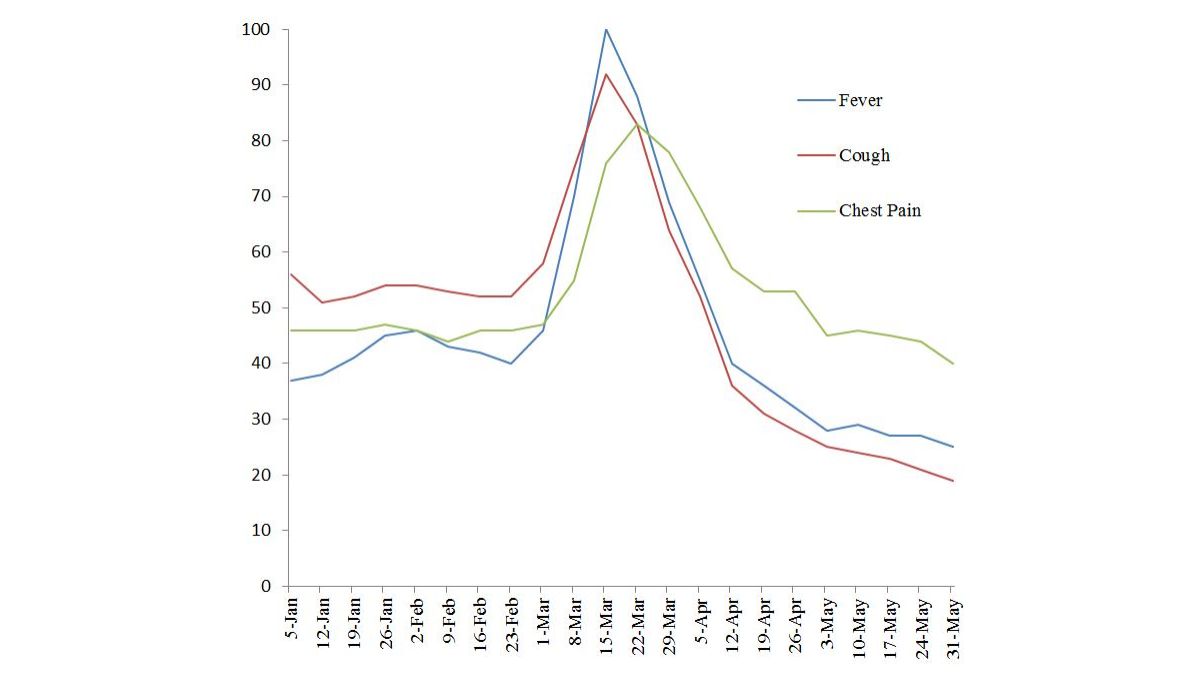
Relative daily search volume of "chest pain," "cough," and "shortness of breath" (January 1, 2020, to May 31, 2020).

The RSV of “chest pain” was compared to “common symptoms of COVID-19,” and this is displayed in Figure 2 from January 1, 2020, to May 31, 2020. All searches had increased in RSV from the period before March 1 to the period after (fever: RSV=30 to 48, *P*=.02; cough: RSV=40 to 44, *P*=.59; chest pain: RSV=43 to 57, *P*=.003); however, throughout March and early April searches for COVID-19 symptoms declined and returned to the previous baseline at an accelerated rate compared to chest pain searches. The search volume for “cough” returned to the pre–COVID-19 baseline on April 12, 2020, while the search volume for “fever” returned to baseline by May 3, 2020. In contrast, the search volume for “chest pain” did not return to its pre–COVID-19 baseline average until May 31, 2020.

**Table 2:** Rising chest pain–related queries from March 15, 2020, to April 15, 2020, United States.

Term		RSV^a^ increase (%)
“**Chest pain”**		
	“covid19 chest pain”	Breakout
	“is chest pain a symptom of COVID-19”	Breakout
	“home remedies for chest pain”	Breakout
	“is chest pain a symptom of the coronavirus”	Breakout
	“natural remedies for chest pain”	Breakout
	“is chest pain a symptom of allergies”	4150
	“COVID chest pain”	1400
“**Chest pain – COVID”**		
	“is chest pain a sign of coronavirus”	Breakout
	“can bad posture cause chest pain”	Breakout
	“persistent pain or pressure in the chest”	Breakout
	“chest pain coronavirus symptom”	600
	“corona chest pain”	550
	“corona virus and chest pain”	450
	“chest pain symptom of coronavirus”	450
“**Chest pain – corona”**		
	“is chest pain a symptom of COVID-19”	Breakout
	“is chest pain a sign of coronavirus”	Breakout
	“chest pain COVID”	1950
	“COVID symptoms”	1450
	“COVID-19 symptoms”	1400
	“chest pain with coronavirus”	1200
	“COVID-19 chest pain”	1200

^a^RSV: relative search volume.

## Discussion

The major finding of our study is the marked increase in search frequency of terms and topics related to chest pain that began at the onset of the COVID-19 pandemic in the four countries studied. The discordance between the rise in chest pain searches and the documented reduction in hospital-recorded ACS admissions and ED presentations raises the possibility that patients chose to avoid presentation to health care facilities despite experiencing concerning cardiac symptoms or attributed such symptoms to another etiology.

During the period of study, searches for chest pain and myocardial infarction occurred with steady frequency until COVID-19 began spreading in the United States. After that time, searches for chest pain increased substantially as may be expected given the known increase in ACS events with other viral illnesses and the social and economic stress presented by the pandemic [12]. However, during the same period, myocardial infarction searches declined. Possibilities for the fall in myocardial infarction searches include a reduced public awareness of myocardial infarction in the context of heightened awareness for COVID-19 [13]. Increased chest pain searches could be due to coronary heart disease or noncardiac causes including COVID-19. The increased frequency in searches such as “is chest pain a symptom of COVID-19” and “coronavirus chest pain” likely indicated some of the increase is from greater interest in COVID-19. However, we evaluated chest pain searches that excluded COVID-19 or corona terms to limit this effect and still found an increase in chest pain search volume supporting a rise in non–COVID-19–related chest pain searches. Several rising related searches seem to reflect people trying to manage symptoms without health care intervention such as “home remedies for chest pain” and natural “remedies for chest pain.”

The rise in the search for COVID-19 symptoms at the onset of the pandemic in the United States is expected; however, these search volumes declined below their baseline averages by early May as opposed to actual infections, which have dramatically increased during that time frame [2]. Chest pain search volume has not declined at the same pace as symptoms for COVID-19 and has remained elevated from baseline through April until the last week of May. If rises in chest pain search volume were solely due to COVID-19–related searches, then its search volume should have mirrored that of publicized symptoms for COVID-19. Interestingly, May represents a month when strict stay-at-home orders were slowly relaxed throughout the country [14]. This could represent a fall in chest pain searches that closely mirrors an increase in people’s ability to seek care.

While there are inherent limitations to infodemiology using Google Trends data, it remains a valuable tool for providing nearly real-time data with extensive search volumes. Once accurate public reporting of ACS admissions incidence is available, it would be useful to compare the changes in search volume seen. Patient survey data regarding their management of chest pain during the epidemic may also provide a useful mechanism to help understand patient decisions. Chest pain has not been reported as a common symptom of COVID-19; however, given the short experience with this pathogen it is possible this could be currently underreported.

Overall, the data provided support for an increase in the burden of chest pain and potentially ACS during the COVID-19 pandemic, a time when multiple reports have shown a drop in ED presentation and admission for ACS. It also provides some insights into strategies patients are using to avoid health care interaction. Public health officials should emphasize and potentially utilize online access to inform the public about the need to seek medical care for potentially life-threatening symptoms even in the midst of the COVID-19 pandemic.

## Abbreviations

ACS: acute coronary syndrome
COVID-19: coronavirus disease
ED: emergency department
RSV: relative search volume
STEMI: ST-elevation myocardial infarction
